# MMP-3 as a predictor for structural remission in RA patients treated with MTX monotherapy

**DOI:** 10.1186/s13075-016-0948-7

**Published:** 2016-02-27

**Authors:** Kazuko Shiozawa, Takashi Yamane, Miki Murata, Ryosuke Yoshihara, Ken Tsumiyama, Shigeaki Imura, Shunichi Shiozawa

**Affiliations:** The Rheumatic Diseases Center, Kohnan Kakogawa Hospital, 1545-1 Saijo, Kannocho, Kakogawa, 675-8545 Japan; Department of Medicine, Rheumatic Diseases Unit, Kyushu University Beppu Hospital, 4546 Tsurumihara, Beppu, 874-0838 Japan

**Keywords:** Rheumatoid arthritis, Radiographic remission, Radiographic progression, Predictor, Matrix metalloproteinase-3, MMP-3, Methotrexate (MTX) monotherapy

## Abstract

**Background:**

The study was undertaken to assess the efficacy of methotrexate (MTX) monotherapy on the radiographic progression of individual rheumatoid arthritis (RA) patients, each of whom had received MTX monotherapy for 3 years with an option to change to biological disease-modifying anti-rheumatic drugs (bDMARDs). We also looked for predictors of radiographic non-progression in these patients.

**Methods:**

Rheumatoid patients (n = 161) were prospectively followed for 3 years while receiving low-dose MTX monotherapy unless disease was otherwise active and/or adverse events appeared. Their disease activity and radiographic progression were evaluated with reference to disease activity score 28 (DAS28), modified health assessment of questionnaire (mHAQ) and other indices. The change in van der Heijde-modified total Sharp score per year (∆TSS) was assessed using probability plots, in which the patients were classified into the subgroups showing structural remission (REM; ∆TSS ≤0.5), radiographic progression (∆TSS >3) or rapid radiographic progression (RRP; ∆TSS >5).

**Results:**

MTX monotherapy, continued until disease became active and/or adverse event appeared, was associated with a significant improvement (p <0.0001) in the DAS28-ESR (3) scores, % DAS28 remission, and mHAQ scores each year, from baseline to 3 years. The mHAQ remission rate (∆mHAQ <0.5) and Boolean remission were also improved from 16 to 60 % and 0.8 to 24.0 %, respectively. We found that the ratio of patients classified as REM increased yearly from 62/161 (38.5 %) to 69/137 (50.4 %), while those classified as ∆TSS >3 decreased from 55/161 (34.2 %) to 28/137 (20.4 %) and those in RRP decreased from 35/161 (21.7 %) to 15/137 (10.9 %). Receiver operating characteristic (ROC) curve analyses showed that serum matrix metalloproteinase-3 (MMP-3) <103.7 ng/ml at outset predicts a patient subgroup that exhibits no radiographic progression.

**Conclusions:**

Half of rheumatoid patients treated with MTX monotherapy for 3 years exhibited structural remission, and this outcome can be predicted at the outset by lower serum MMP-3.

**Electronic supplementary material:**

The online version of this article (doi:10.1186/s13075-016-0948-7) contains supplementary material, which is available to authorized users.

## Background

Methotrexate (MTX) has been recommended not only as a first-line drug for the initial treatment of rheumatoid arthritis (RA), but also as an essential component of combination therapies utilizing either conventional disease-modifying anti-rheumatic drugs (cDMARDs) or biological DMARDs (bDMARDs) [1, 2]. Recent studies have shown that patients treated with a combination therapy of MTX and bDMARDs fare better than those given MTX alone [3–18]. However, some individual patients have been shown to respond well to MTX monotherapy, i.e., they exhibit no radiographic progression.

Recently, O’Dell et al. have shown that treating patients with MTX monotherapy initially, while later providing an option to step up to combination therapy produces outcomes similar to those seen with combination therapies consisting of cDMARDS and/or bDMARDs that are provided from the outset [19]. In their study, approximately 30 % of the patients treated by MTX monotherapy did not require subsequent combination therapy. However, this subgroup was reported to be clinically and radiographically indistinguishable from those who required it.

For MTX monotherapy to be more effectively employed as a first-line drug to halt radiographic progression. it would be useful at outset to ascertain which patients would benefit most from MTX monotherapy and which would require combination therapy including biologic agents. The purpose of the present study was to assess the efficacy of low-dose MTX monotherapy, a regimen that is commonly prescribed in Japan, and to potentially identify a subgroup of patients on MTX monotherapy, without radiographic evidence of disease progression. In the present study, 161 patients with rheumatoid arthritis were followed unless disease was otherwise active or significant adverse events appeared. Disease progression was scored as the change in the modified total Sharp score per year (∆TSS), starting at baseline and continuing for 3 years. Patients were classified into subgroups exhibiting structural remission (REM; ∆TSS ≤0.5), radiographic evidence of progression (∆TSS >3) or radiographic evidence of rapid progression (RRP; ∆TSS >5) [20].

## Methods

### Patients and the study design

Patients were included who had RA (n = 161) as determined by the 1987 American College of Rheumatology (ACR) criteria [21] and who had started MTX monotherapy for the first time between January 2005 and August 2010 (Table 1). Patient consent was in accordance with protocols approved by the respective institutional ethical committees of Kohnan Hospital (protocol H16.11.9) and Kyushu University (protocol 875). Only patients who underwent MTX monotherapy for at least 7 months were included, and they were prospectively followed for 3 years while receiving low-dose MTX monotherapy, unless disease was otherwise active and/or adverse events appeared. The dose of MTX used was allowed to vary according to disease activity. Patients who had already been receiving low-dose prednisolone (mean 5, median 5, range 2–10) per day were included and allowed to continue this dosing (Table 1), whereas patients who were just starting prednisolone were excluded. Exclusion criteria were disease activity score in 28 joints-erythrocyte sedimentation rate (DAS28-ESR) >4.2 and clinical disease activity index (CDAI) >22, and/or the emergence of a significant adverse event: in these cases, additional therapies, including bDMARDs, were initiated. Patients were also assessed using the 2010 ACR/European League Against Rheumatism (EULAR) criteria [22].

**Table 1:** Profile of patients at baseline

Patients, number	161		
Male/female, number	40/121		
Age, years	57.4 ± 12.2	(median 58)	n = 161
Onset age, years	53.0 ± 12.4	(median 54)	n = 161
Disease duration, years	4.4 ± 6.9	(median 1.4)	n = 161
Methotrexate used, number of patients (%)	161 (100)		n = 161
Baseline methotrexate dose, mg/wk	4.3 ± 0.9	(median 4)	n = 161
Prednisolone used, number (%)	38 (23.6)		n = 38
Baseline prednisolone dose, mg/day	5.0 ± 3.2		n = 38
Baseline prednisolone dose, mg/day	1.2 ± 2.6	(median 0)	n = 161
Conventional disease-modifying anti-rheumatic drugs used, number of patients (%)	73 (45.3)		n = 161
Stage 1	73	45.3 %	
Stage 2	42	26.1 %	
Stage 3	28	17.4 %	
Stage 4	18	11.2 %	
Class I	78	48.4 %	
Class II	76	47.2 %	
Class III	7	4.3 %	
Class IV	0	0.0 %	
C-reactive protein, mg/dl	2.6 ± 3.0	(median 1.5)	n = 161
Erythrocyte sedimentation rate, mm/h	55.1 ± 35.1	(median 48)	n = 161
Tender joint count	7.1 ± 5.7	(median 6)	n = 161
Swollen joint count	7.5 ± 5.4	(median 7)	n = 161
Disease activity score in 28 joints-C-reactive protein (4)	4.8 ± 1.1	(median 4.6)	n = 129
Disease activity score in 28 joints-erythrocyte sedimentation rate (4)	5.5 ± 1.2	(median 5.5)	n = 129
Disease activity score in 28 joints-C-reactive protein (3)	4.5 ± 1.0	(median 4.3)	n = 161
Disease activity score in 28 joints-erythrocyte sedimentation rate (3)	5.2 ± 1.1	(median 5.2)	n = 161
Visual analog scale, mm	52.4 ± 28.0	(median 51)	n = 129
Matrix metalloproteinase-3, ng/ml	241.9 ± 304.2	(median 133)	n = 157
Rheumatoid factor	119.2 ± 226.5	(median 45.3)	n = 157
Morning stiffness, minutes	165.6 ± 362.6	(median 35)	n = 158
Grip strength, (L+ R)/2 mmHg	176.8 ± 66.7	(median 171)	n = 157
Modified health assessment questionnaire	0.543 ± 0.469	(median 0.500)	n = 130
Baseline van der Heijde modified total Sharp score	18.6 ± 33.2	(median 4.0)	n = 161
Baseline van der Heijde modified total Sharp score, progression, –1 to 0 years	7.9 ± 19.0	(median 3.0)	n = 161

### Assessment

Disease activity was evaluated by joint counts, visual analog scale (VAS), morning stiffness, grip strength, C-reactive protein (CRP), erythrocyte sedimentation rate (ESR), rheumatoid factor (RF), matrix metalloproteinase-3 (MMP-3), anti-citrullinated protein antibody (ACPA), disease activity score in 28 joints (DAS28), and the modified health assessment questionnaire (mHAQ). Patients were followed prospectively for 3 years until cessation of MTX monotherapy. Incomplete data from patients who had dropped out because of our exclusion criteria (n = 25) were assessed in two ways: (1) clinical endpoints were imputed as the last observation carried forward (LOCF) and (2) clinical endpoints were assessed as observed. We obtained similar results with both methods. We also followed the sequelae of patients who had dropped out, and observations that were applicable were described in the results. Radiographic evidence of progression was assessed in the hands and wrists and scored chronologically as recommended by Bruynesteyn et al. [23] using the TSS as previously described [24]. The change in TSS per year was evaluated from the baseline and annual evaluation of patients, and recorded as the TSS year-progression (ΔTSS). From these data, patients were classified into three groups: those with structural remission (REM) (ΔTSS ≤0.5), those with radiographic evidence of progression (ΔTSS >3), and those with radiographic evidence of rapid progression (RRP) (ΔTSS >5) [22]. Baseline and annual data were assessed in relation to radiographic evidence of progression to identify a factor that could predict which subgroup of patients may respond to treatment, i.e., patients who showed no radiographic evidence of progression, and did not fall into either the ∆TSS >3 or RRP (∆TSS >5) classification.

### Statistical analysis

Radiographic evidence of progression and functional outcomes over time were compared using the Wilcoxon signed-rank test. Demographic and baseline characteristics were analyzed using Fisher’s exact test for categorical variables and the Wilcoxon rank sum test for continuous variables. Receiver operating characteristics (ROC) curve analysis was conducted using MMP-3 protein levels, which had been identified as a potential predictor of disease, using univariate and multivariate analysis to determine the cutoff value for a diagnostic test of joint damage. Multivariate analysis was performed using multiple regression models with variables for which the *p* values were <0.2 in the preceding univariate analysis. All reported *p* values are two-sided and not adjusted for multiple testing. Any difference with a *p* value <0.05 was considered statistically significant. All analyses were performed using Statview for Windows V.5.0 (SAS Institute, Cary, NC, USA), R version 2.15.2, and the Epi library.

## Results

### Effect of low-dose MTX monotherapy on disease activity

All patients who were included fulfilled the 2010 ACR/EULAR criteria [24] and exhibited high disease activity (mean ± SD for DAS28-ESR (3), 5.2 ± 1.1; for CRP, 2.6 ± 3.0 mg/dL) and progressive joint destruction (mean ± SD for baseline TSS, 18.6 ± 33.2; for baseline ∆TSS for –1 to 0 years, 7.9 ± 19.0) at the time of initiation of MTX monotherapy (Table 1). Low doses of MTX were used in this study (mean ± SD, median: 4.3 ± 0.9 mg/wk, 4 mg/wk at baseline; 6.7 ± 2.5 mg/wk, 6 mg/wk after 1 year; 7.0 ± 2.7 mg/wk, 6 mg/wk after 2 years; 6.8 ± 2.7 mg/wk, 6 mg/wk after 3 years). Prednisolone was prescribed to 38 of 161 patients (23.6 %), with a mean ± SD dose of 5.0 ± 3.2 mg/day at baseline, 4.2 ± 2.4 mg/day after 1 year, 3.7 ± 2.0 mg/day after 2 years, and 3.5 ± 2.1 mg/day after 3 years, respectively.

**Fig. 1:**
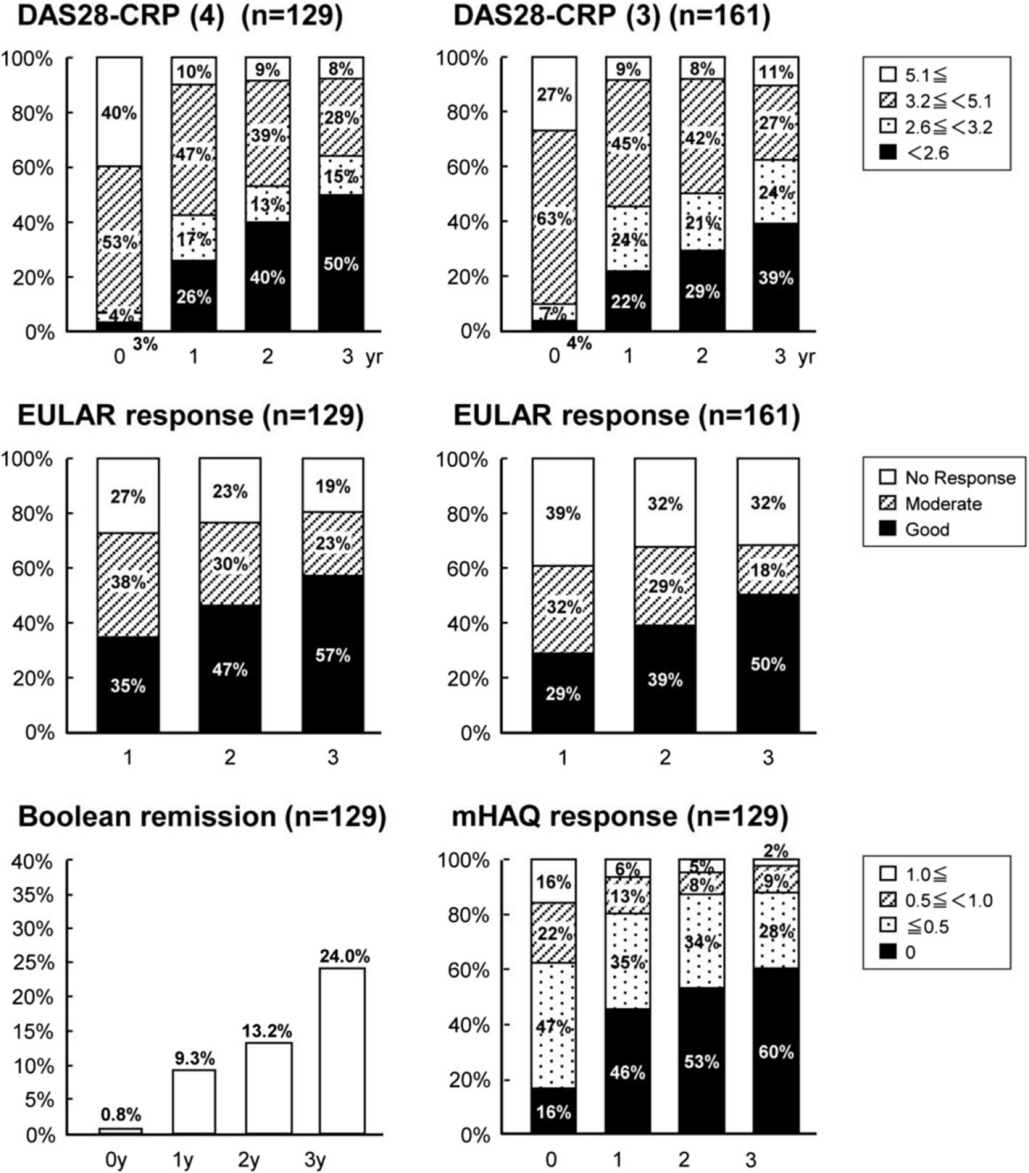
Annual changes in disease activity score in 28 joints (*DAS28*)-C-reactive protein (*CRP*), European League Against Rheumatism (*EULAR*) response, modified health assessment questionnaire (*mHAQ*) and Boolean remission rate

Patients with rheumatoid arthritis (n = 161) were prospectively followed for 3 years while receiving low-dose MTX monotherapy unless disease was otherwise active and/or adverse events appeared. Disease activity was found to be significantly improved each year starting at baseline and continuing to 3 years: DAS28-ESR (3) decreased from 5.2 ± 1.1 to 3.9 ± 1.4 (*p* <0.0001) and DAS28-CRP (3) from 4.5 ± 1.0 to 3.1 ± 1.3 (*p* <0.0001) (Table 2). The percent DAS28-ESR (3) remission increased from 1 to 19 % (Additional file 1: Figure S1), and the percent DAS28-CRP (3) remission increased from 4 to 39 % (Fig. 1). The EULAR responses were also improved. The mHAQ improved from 0.54 ± 0.47 to 0.18 ± 0.32 (*p* <0.0001) (Table 2), and the mHAQ remission rate (ΔmHAQ <0.5) from 16 to 60 % (Fig. 1). The ratio of patients who achieved Boolean remission also increased from 0.8 to 24.0 % during this 3-year period (Fig. 1). Similar results were obtained when data were analyzed as observed instead of by the LOCF (data not shown).

**Table 2:** Annual changes in disease activity indices and serum MMP-3 levels

	0 year	1 year	2 years	3 years	Number of patients
DAS28-ESR(4)	5.5 ± 1.2	4.1 ± 1.3***	3.8 ± 1.3***	3.6 ± 1.4***	129
DAS28-CRP(4)	4.8 ± 1.1	3.4 ± 1.2***	3.1 ± 1.2***	2.6 ± 1.3***	129
DAS28-ESR(3)	5.2 ± 1.1	4.2 ± 1.3***	4.0 ± 1.23***	3.9 ± 1.4***	161
DAS28-CRP(3)	4.5 ± 1.0	3.5 ± 1.2***	3.2 ± 1.2***	3.1 ± 1.3***	161
mHAQ	0.541 ± 0.470	0.269 ± 0.388***	0.205 ± 0.318***	0.180 ± 0.323***	129
MMP3, ng/ml	241.9 ± 304.2	178.5 ± 287.8**	174.7 ± 299.5***	181.5 ± 321.2***	157

**p = 0.0015, ***p <0.0001 vs 0 year analyzed by the Wilcoxon signed-rank test. *DAS28* disease activity score in 28 joints, *ESR* erythrocyte sedimentation rate, *CRP* C-reactive protein, *mHAQ* modified health assessment questionnaire, *MMP* matrix metalloproteinase-3

### Effect of low-dose MTX monotherapy on radiographic evidence of progression

**Fig. 2:**
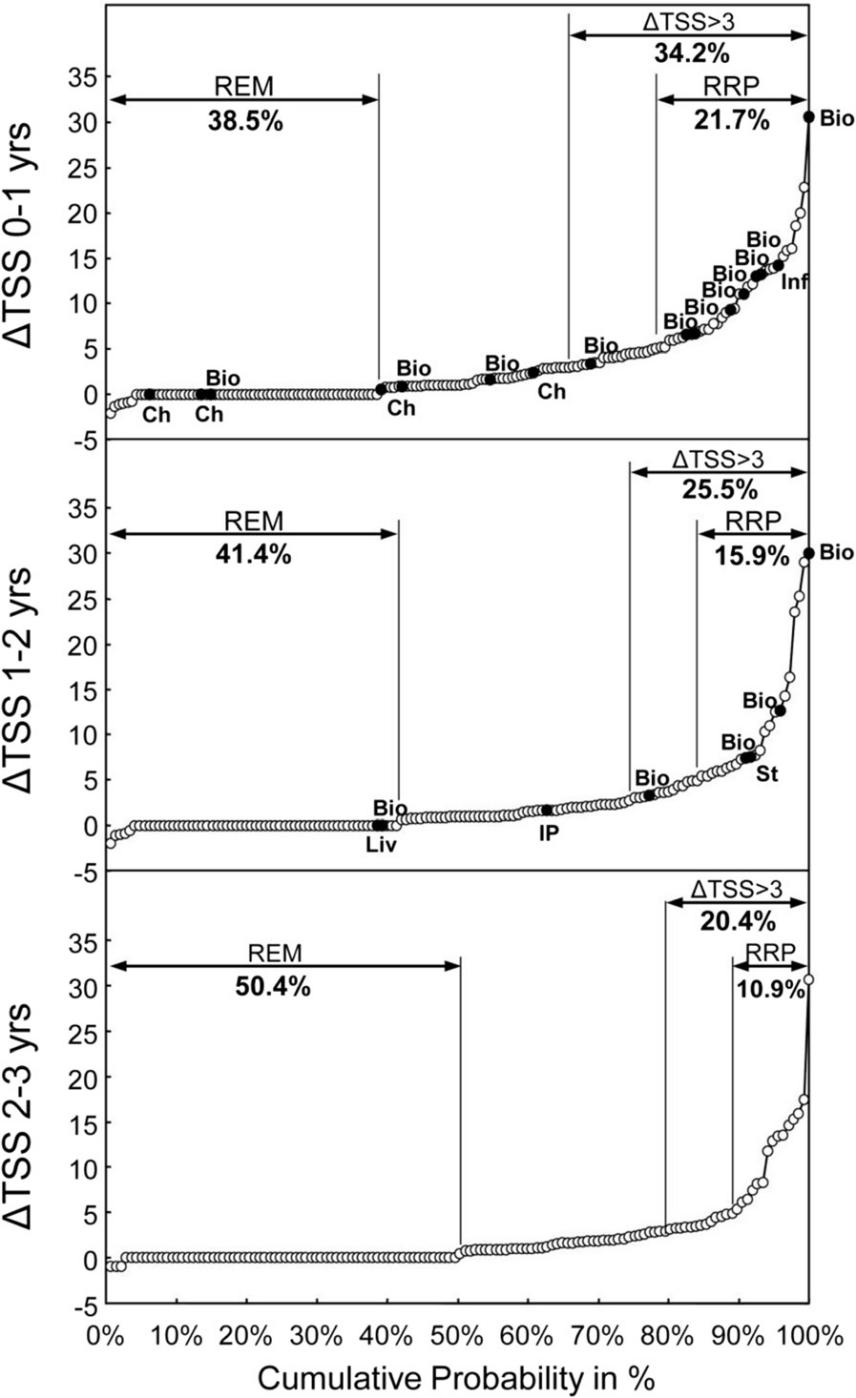
Cumulative probability plots for the change in van der Heijde modified total Sharp score per year (*ΔTSS*). Patient subgroups corresponding to structural remission (*REM*), radiographic evidence of progression (ΔTSS >3), or radiographic evidence of rapid progression (*RRP*) are indicated. *Bio* indicates a patient who began new treatment with a biologic agent. *Ch* indicates a patient who moved to another hospital because of inactive disease but continued methotrexate (MTX) treatment. *Inf* indicates a patient who developed infection, mandibular myelitis, and thus discontinued MTX treatment. *Liv* indicates a patient who had abnormal liver tests and thus discontinued MTX treatment. *IP* indicates a patient who developed interstitial pneumonitis and thus discontinued MTX treatment. *St* indicates a patient who developed stomatitis and thus discontinued MTX treatment

Radiographic evidence of progression as determined by mean ∆TSS significantly decreased over the 3-year study period. Respective measurements (mean ± SD (n), median, statistical significance) were as follows: ∆TSS 0–1 year, 3.3 ± 5.1 (n = 161), 1.0, *p* <0.0001; ∆TSS 1–2 years, 2.7 ± 5.2 (n = 145), 1.0, *p* <0.0001; and ∆TSS 2–3 years, 2.2 ± 4.4 (n = 137), 0.5, *p* <0.0001. Cumulative probability plots showed that after 1 year of MTX monotherapy, 62/161 of the patients (38.5 %) were classified into the REM category, 55/161 (34.2 %) into ∆TSS >3, and 35/161 (21.7 %) into RRP (Fig. 2, upper). Among the 62 patients in the REM category, 2 (3.2 %) were clinically stable and moved to another hospital: subsequent follow up showed that these patients had inactive disease for the remaining 2 years. One other patient (1.6 %) with clinically active disease had MTX monotherapy stopped and infliximab (IFX) therapy initiated because the patient had a DAS28-ESR (3) >3.2. Among the 20 patients in the ∆TSS >3 but not RRP category, 3 patients (15.0 %) had active disease and thus IFX therapy was added. Two patients (10.0 %) were stable and moved to another hospital: subsequent follow up showed that they had inactive disease for the remaining 2 years. Among the 35 patients in the RRP category, 8 patients (22.9 %) had active disease and biologic agents were added to their regimen. One of these patients developed mandibular myelitis and MTX therapy was stopped for 4 months and then resumed.

During the second year of MTX monotherapy (years 1–2), 60/145 patients (41.4 %) were classified as REM, 37/145 (25.5 %) as ∆TSS >3, and 23/145 (15.9 %) as RRP (Fig. 2, middle). Among the 60 patients classified as REM, one patient (0.017 %) showed signs of liver injury and thus MTX therapy was stopped and tocilizumab (TCZ) was initiated. IFX was initiated in one other patient (0.017 %) due to the patient’s personal preference, although the disease was inactive. Among the 14 patients classified as ∆TSS >3 but not RRP, disease became clinically active in one patient (7.1 %) and thus biologic agents were added. Another patient (with ∆TSS ≤3) developed interstitial pneumonitis and thus MTX was stopped and tacrolimus was initiated. Among the 23 patients with RRP, disease became clinically active in 3 (11.3 %) and thus biologic agents were added. One other patient (0.04 %) developed stomatitis and thus MTX therapy was stopped.

During the third year of MTX monotherapy (years 2–3), 69/137 patients (50.4 %) were classified as REM, 28/137 (20.4 %) as ∆TSS >3, and 15/137 (10.9 %) as RRP (Fig. 2, lower). These data indicate that over time, patients with active disease who required biologic agents were mostly in the RRP group, followed by the ∆TSS >3 group. This was not the case, however, when the patients were classified according to disease activity, i.e., DAS28 (Additional file 1: Table S1).

In summary, during the 3 years of MTX monotherapy, the percentage of patients classified into the REM group increased from 38.5 to 50.4 % (*p* = 0.0466), those in ∆TSS >3 decreased from 34.2 to 20.4 % (*p* = 0.0095), and those in RRP decreased from 21.7 to 10.9 % (*p* = 0.0190) (Fig. 2).

### Search for a factor that predicts which patients will show no radiographic evidence of progression with MTX monotherapy

**Fig. 3:**
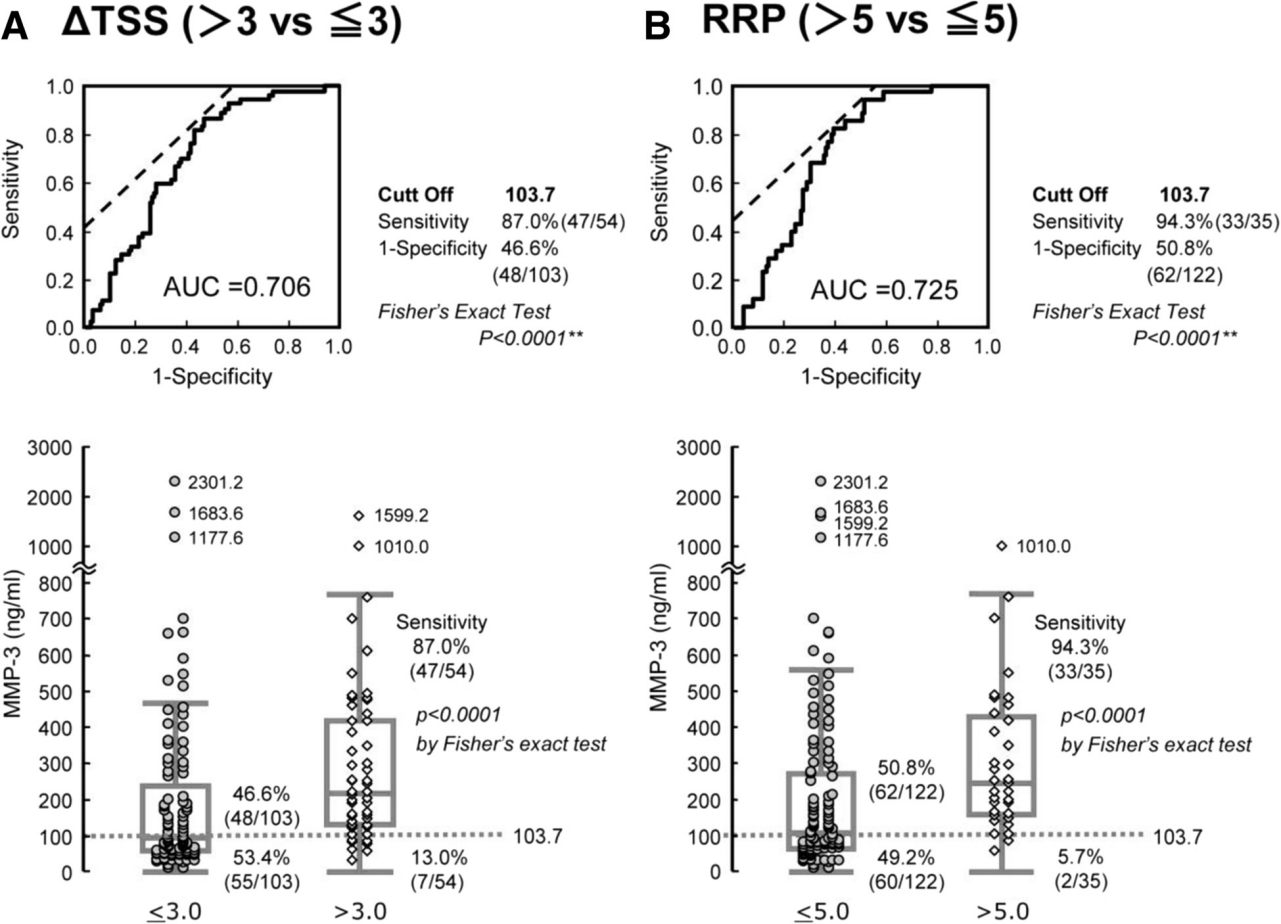
Receiver operating characteristics curve analysis of **a** radiographic evidence of progression (van der Heijde modified total Sharp score year-progression (*ΔTSS*) >3)) and **b** radiographic evidence of rapid progression (*RRP*), showing that serum matrix metalloproteinase-3 (*MMP-3*) levels measured at the outset of methotrexate monotherapy can predict radiographic evidence of non-progression of disease. *AUC* area under the curve

**Fig. 4:**
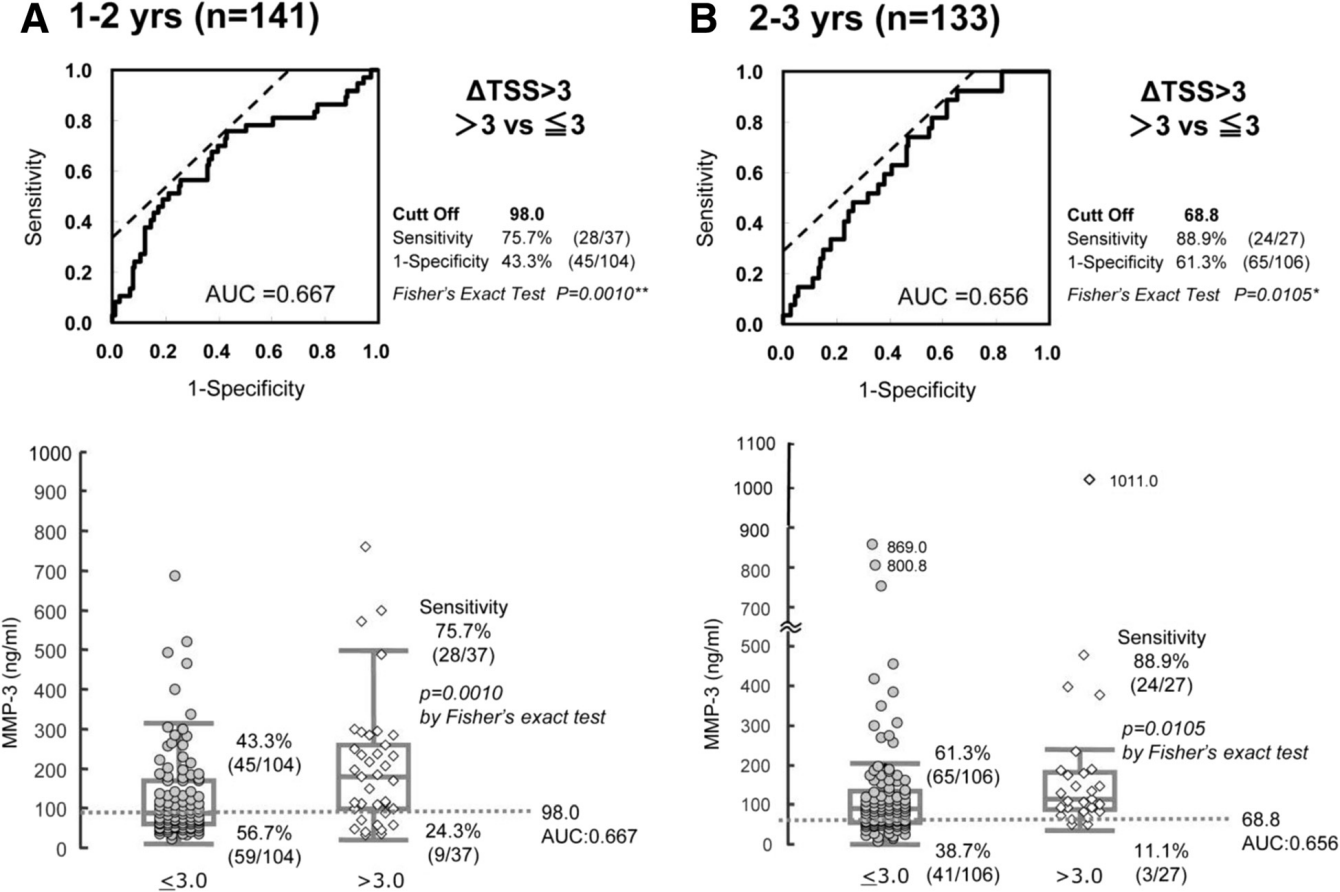
Receiver operating characteristics curve analysis of radiographic progression (van der Heijde modified total Sharp score year-progression (*ΔTSS*) >3 versus ΔTSS ≤3, showing that serum matrix metalloproteinase-3 (*MMP-3*) levels of serum MMP-3 as measured at 1 year (**a**) or 2 years (**b**) after the start of methotrexate monotherapy can predict final radiographic evidence of non-progression of disease. *AUC* area under the curve

We searched for a factor that might identify a subgroup of patients who did not have radiographic evidence of progression as assessed by ROC curve analysis. We found that patients with serum MMP-3 levels <103.7 ng/ml at baseline came to be classified in neither the ∆TSS >3 nor the RRP groups, with negative predictive values (NPV) of 88.7 and 96.8 %, respectively (Fig. 3). This cutoff level of 103.7 ng/ml was valid when male and female patients were studied separately, with an NPV of 88.1 % for ∆TSS >3 and 87.8 % for RRP, respectively (Additional file 1: Figure S2). We also noted that as the clinical course of therapy proceeded, lower serum MMP-3 levels predicted a subgroup that exhibited no radiographic evidence of progression: 98.0 ng/ml as measured after 1 year of MTX monotherapy with an NPV of 86.8 % for this 1–2 year period, and 68.8 ng/ml after 2 years of MTX monotherapy, with NPV of 93.2 % for the 2–3 year period (Fig. 4).

## Discussion

Initial application of MTX monotherapy followed by combination therapy with bDMARDs, if necessary, is a reasonable therapeutic strategy for treating patients with early RA [25]. In the present study, we found that a substantial proportion of patients with RA fared well with just MTX monotherapy: they did not exhibit radiographic evidence of progression and did not require combination therapy. This result was in part due to the careful monitoring of patients and to the availability of other treatment options to individuals who exhibited active disease and/or adverse events. Nevertheless, it was somewhat surprising to find that approximately half of the patients who would have been considered to have a poor prognosis as assessed by the EULAR/ACR criteria [22] nevertheless had no radiographic evidence of progression for 3 years while being treated only with low-dose MTX monotherapy. The dose of MTX used was significantly lower than those used in other countries, due to national healthcare regulations unique to Japan at that time [26]. We considered the possibility that genetic factors unique to the Japanese population might underlie the high efficacy of this low-dose MTX monotherapy. However, our findings are consistent with those of O’Dell et al. [19] who reported that approximately 30 % of Caucasian patients fared well with MTX monotherapy and did not require combination therapy. The patients in our studies may be more typical of the patients encountered by clinicians in daily practice compared to those in the O’Dell study, as most patients had a significantly longer history of disease prior to entering the study (average 4.4 years). Many of these patients had previous exposure to other therapies including sulfasalazine (36 %) or bucillamine (38 %). Our observations may more closely represent the outcomes to be expected with low-dose MTX monotherapy in a typical real-life patient population, and indicates that MTX monotherapy may be useful as a first-line drug to halt radiographic evidence of progression in RA.

As to which patients might benefit most from MTX monotherapy and which would require additional therapy with biologic agents or others to halt progression as seen on radiography, we found that low serum MMP-3 measured at the outset of MTX monotherapy, was a good predictor of radiographic non-progression. We further noted that lower serum MMP-3 measured during the course of MTX monotherapy is also a good predictor. The results are in line with the finding of Ma et al., who found that continuously elevated serum MMP-3 predicts radiographic evidence of progression for 1 year in the patients treated with various cDMARDs in a treat-to-target (T2T) protocol [27]. Previous studies have shown that joint destruction is halted if all of the joint-destroying MMPs including MMP-3, MMP-9 and MMP-13 are normalized [28, 29]. Although serum MMP-3 levels alone are a crude reflection of disease progression [30], they presumably reflect the levels of other joint-destroying MMPs as well, as for example shown in the collagen-induced arthritis model in mice [31]. These may explain why serum MMP-3, as determined by ROC curve analysis, is negatively correlated with later radiographic evidence of progression in the present study.

We also noted that clinically active disease emerged primarily in patients in the RRP group. Patients classified as REM or ∆TSS ≤3 seldom developed clinically active disease during the course of MTX monotherapy, and this was especially true among patients who remained in REM for more than 1 year of MTX monotherapy. These results support the rationale for the use of MTX monotherapy as a first choice to halt radiographic evidence of progression, unless or until disease becomes active and/or adverse events appear. This rationale is further supported by recent studies that suggest that MTX may even extend the life expectancy of patients with rheumatoid disease [32, 33].

## Conclusions

In conclusion, we found that structural remission and radiographic evidence of non-progression were respectively induced in 50.4 and 79.6 % of patients with rheumatoid disease treated continuously with MTX monotherapy, with an option to change to bDMARDs for 3 years. We also found that low serum MMP-3 measured at the outset of MTX monotherapy can be a good predictor of radiographic evidence of non-progression.

## Additional file

Additional file 1: Figure S1. Annual changes in disease activity score in 28 joints-erythrocyte sedimentation rate (DAS28-ESR) and European League Against Rheumatism (EULAR) response. **Figure S2.** Receiver operating characteristic (ROC) curve analysis showing that serum matrix metalloproteinase-3 (MMP-3) levels measured at the outset of methotrexate (MTX) monotherapy can predict radiographic evidence of non-progression in male (**a**) and female (**b**) patients, respectively. **Table S1.** Relationship between disease activity score (DAS) and van der Heijde modified total Sharp score year-progression (∆TSS). (DOCX 1416 kb)

## Abbreviations

ACR: American College of Rheumatology
bDMARD: biological disease-modifying anti-rheumatic drug
CDAI: clinical disease activity index
cDMARD: conventional disease-modifying anti-rheumatic drug
CRP: C-reactive protein
DAS: disease activity score
DMARD: disease-modifying anti-rheumatic drug
ESR: erythrocyte sedimentation rate
EULAR: European League Against Rheumatism
IFX: infliximab
LOCF: last observation carried forward
mHAQ: modified health assessment questionnaire
MMP: matrix metalloproteinase
MTX: methotrexate
NPV: negative predictive value
REM: structural remission
RF: rheumatoid factor
ROC: receiver operating characteristic
RRP: rapid radiographic progression
TCZ: tocilizumab
TSS: van der Heijde modified total Sharp score
VAS: visual analog scale
∆TSS: TSS van der Heijde modified total Sharp score year-progression
