# Supplementary figures and images for: MMP-3 as a predictor for structural remission in RA patients treated with MTX monotherapy
Source: Arthritis Res Ther. 2016 Feb 27;18:55. doi: 10.1186/s13075-016-0948-7 (PMC4769545; doi:10.1186/s13075-016-0948-7)

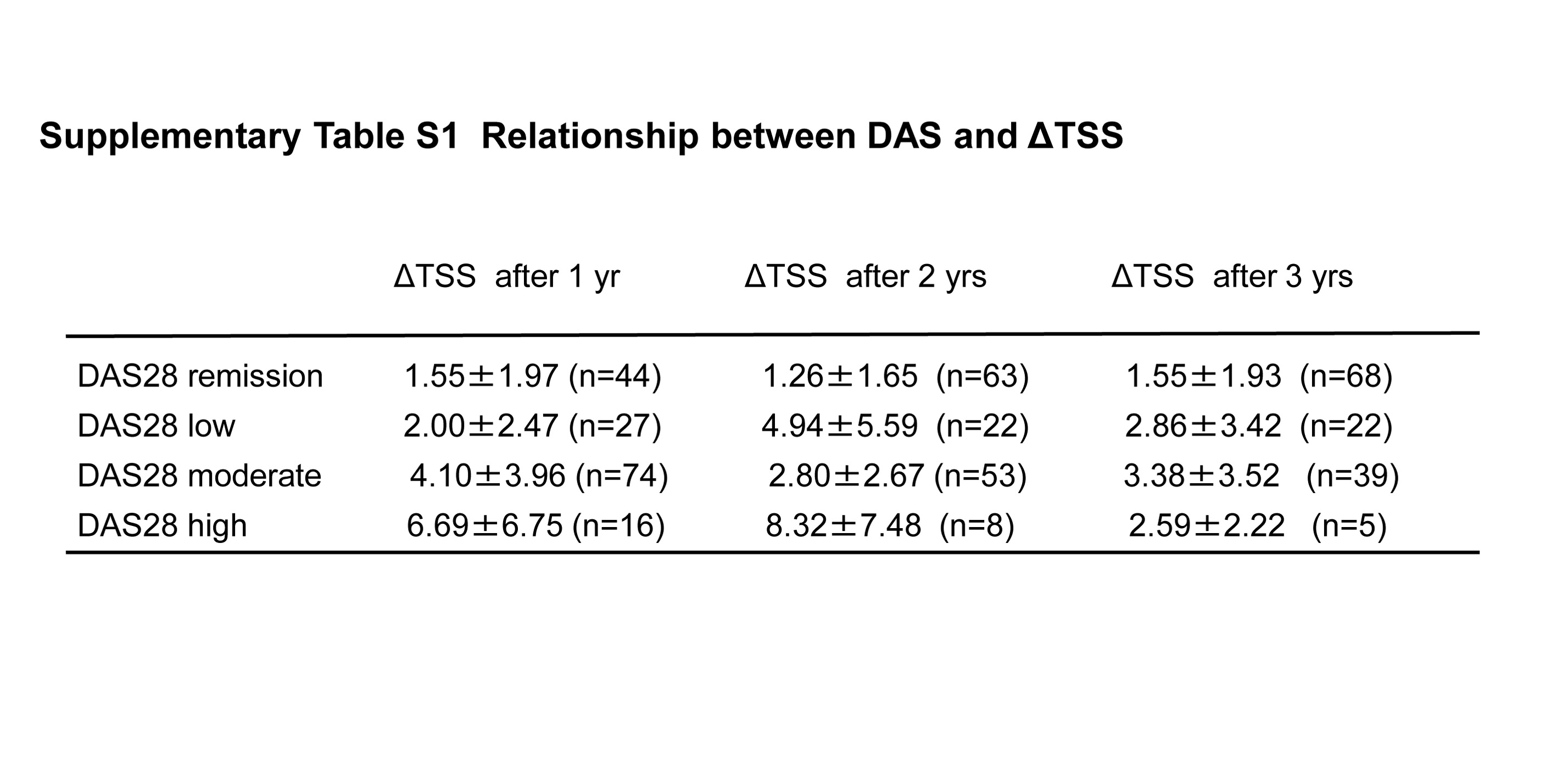


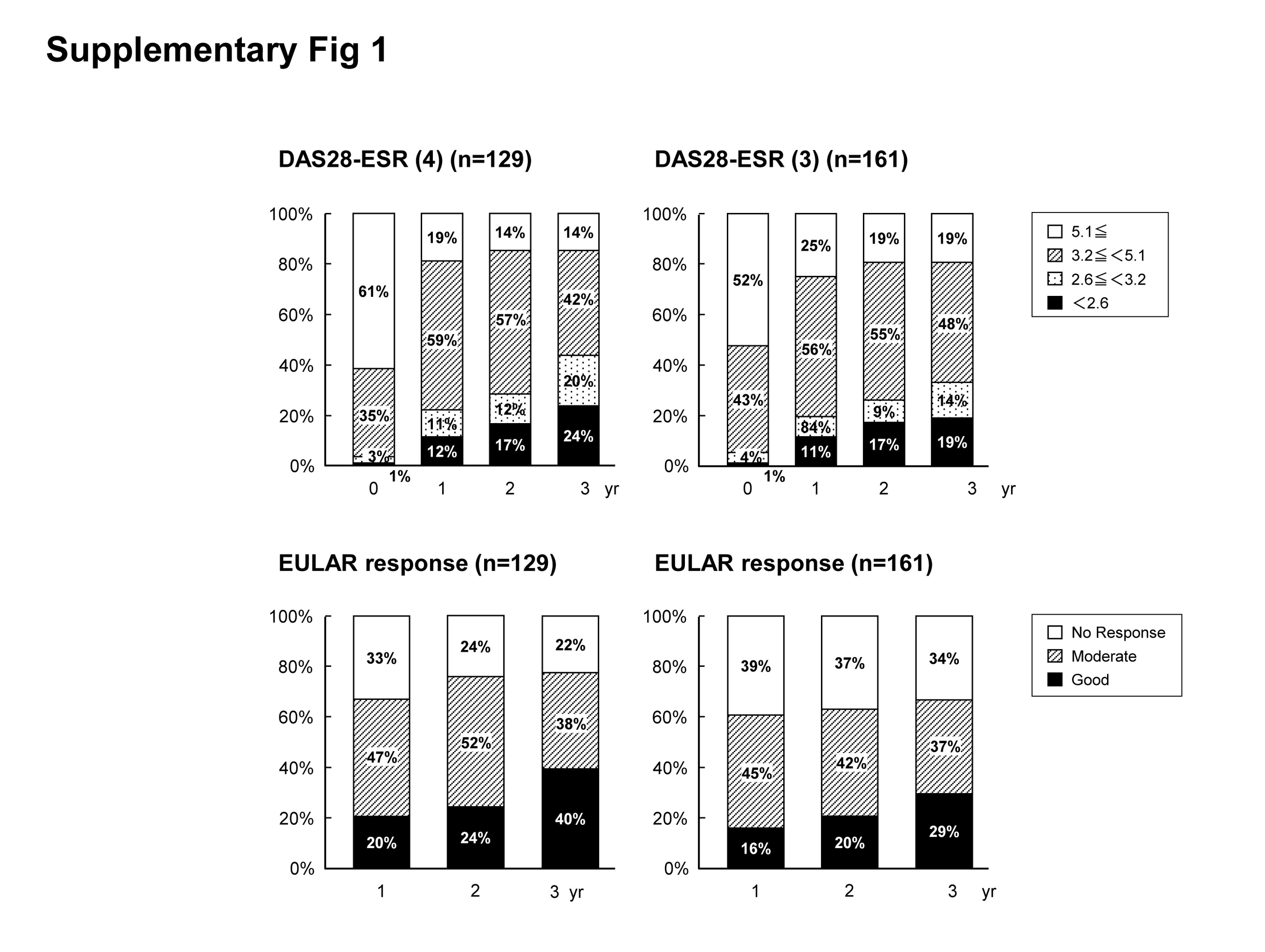


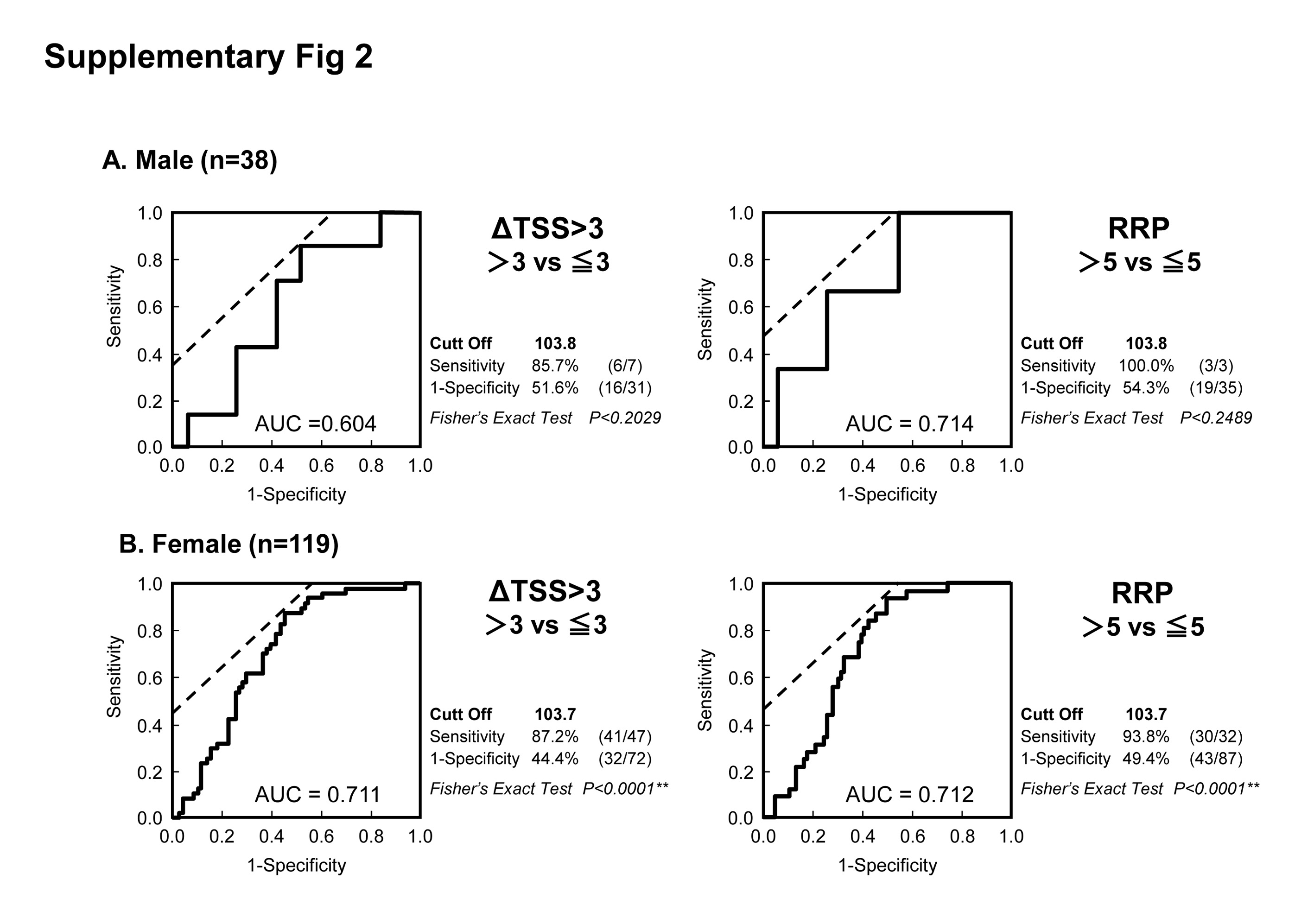

Supplement: Additional file 1: Figure S1. — Annual changes in disease activity score in 28 joints-erythrocyte sedimentation rate (DAS28-ESR) and European League Against Rheumatism (EULAR) response. Figure S2. Receiver operating characteristic (ROC) curve analysis showing that serum matrix metalloproteinase-3 (MMP-3) levels measured at the outset of methotrexate (MTX) monotherapy can predict radiographic evidence of non-progression in male (a) and female (b) patients, respectively. Table S1. Relationship between disease activity score (DAS) and van der Heijde modified total Sharp score year-progression (∆TSS). (DOCX 1416 kb) [file 13075_2016_948_MOESM1_ESM.docx]
